# ADAMTS Expression in Colorectal Cancer

**DOI:** 10.1371/journal.pone.0121209

**Published:** 2015-03-18

**Authors:** Serafula Filou, Aggeliki Korpetinou, Dora Kyriakopoulou, Dimitrios Bounias, Michael Stavropoulos, Panagiota Ravazoula, Dionysios J. Papachristou, Achilleas D. Theocharis, Demitrios H. Vynios

**Affiliations:** 1 Biochemistry, Biochemical Analysis and Matrix Pathobiology Research Group, Laboratory of Biochemistry, Department of Chemistry, University of Patras, Patras, Greece; 2 Department of Surgery, School of Health Sciences and University Hospital of Patras, Patras, Greece; 3 Department of Histopathology, University Hospital of Patras, Patras, Greece; 4 Anatomy, Histology and Embryology laboratory, Department of Medicine, University of Patras, Patras, Greece; University of Insubria, ITALY

## Abstract

ADAMTSs are a family of secreted proteinases that share the metalloproteinase domain with matrix metalloproteinases (MMPs). By acting on a large panel of extracellular substrates, they control several cell functions such as fusion, adhesion, proliferation and migration. Through their thrombospondin motifs they also possess anti-angiogenic properties. We investigated whether ADAMTSs participate in colorectal cancer progression and invasion. Their expression was investigated at both mRNA and protein levels. Using RT-PCR, the expression of *ADAMTS-1*, *-4*, *-5* and *ADAMTS-20* was estimated in colorectal tumors of different cancer stage and anatomic site and 3 cell lines of different aggressiveness. An overexpression of ADAMTS-4 and -5 was observed, especially in tissue samples, whereas ADAMTS-1 and -20 were found to be down-regulated. Western blot analysis further supported the RT-PCR findings, revealing in addition the degradation of ADAMTS-1 and -20 in cancer. In situ expression and localization of *ADAMTS-1*, *-4*, *-5* and *-20* was also investigated by immunohistochemical analysis. Our data suggest a positive correlation between *ADAMTS-4* and *-5* expression and cancer progression, in contrast with the anti-angiogenic members of the family, *ADAMTS-1* and *-20*, which were found to be down-regulated. Our findings support the notion that overexpression of ADAMTS-4 and ADAMTS-5 in colorectal cancer might be a possible invasive mechanism of cancer cells in order to degrade proteoglycans of ECM.

## Introduction

Colorectal cancer (CRC) is the third most frequently diagnosed cancer and the second leading cause of cancer deaths in the United States [1] and Europe, as well. Although CRC incidence, in economically developed countries, is stabilized or rather declined [2], there is still the question for a greater understanding of the biology of angiogenesis and invasion with a view to the discovery of antiangiogenic/anti-invasive molecules. Accumulating evidence demonstrates the crucial role of proteolytic enzymes such as matrix metalloproteinases (MMPs) and closely related ADAMTSs (a disintegrin and metalloproteinase with thrombospondin motif) in cancer development and progression [3]. ADAMTS are a distinct group of zinc-dependent metalloproteinases and display sequence similarities with the reprolysin family of snake venomases. The complete human ADAMTS family comprises 19 genes [4] (ADAMTS-5 and ADAMTS-11 share the same gene) and although they are soluble proteins, many of them appear to bind the extracellular matrix through their thrombospondin motifs or their spacer region [5–7]. ADAMTS have varying functions, including specific cleavage of the matrix proteoglycans aggrecan, versican and brevican (ADAMTS-1, -4, -5, -8 and -15) [7–9], inhibition of angiogenesis (ADAMTS-1, -8) [8–12] and collagen processing (ADAMTS-2, -3 and -14) [13].

ADAMTS-1 and -8 are considered to be anti-angiogenic factors, because of the interaction between their thrombospondin motifs and CD36, a membrane glycoprotein receptor of endothelial cells [11, 12]. These proteases have been shown to inhibit VEGF-induced angiogenesis in the chorioallantoic membrane (CAM) assay [10] and suppress FGF-2-induced vascularisation in the cornea pocket assay. However, ADAMTS-1 should be considered as a molecule with anti- or pro-metastatic effects depending on the cleavage site, during its auto-proteolytic cleavage [14]. ADAMTS-8, seems also to inhibit epidermal growth factor receptor signaling along with decreased levels of phosphorylated MEK and ERK [15]. Moreover, the 12q12 gene locus of ADAMTS-20 has been found to be subject to translocations and other alterations in human malignancies [16–19] and also several pathologic conditions, including Parkinson’s disease [20].

There is a wealth of evidence demonstrating that ADAMTS are deregulated in human cancer. Indeed, previous studies have shown that ADAMTS-20 is overexpressed in brain and breast carcinomas, suggesting that this protease could play a role in tumor progression. For ADAMTS-1, it has been shown that overexpression of its full-length isoform enhances tumor growth and promotes pulmonary metastasis of TA3 mammary cells or Lewis lung cells, while it has been found decreased in non-small cell lung carcinoma, pancreatic tumors and prostate cancer cell lines [21, 22]. It has also been shown that ADAMTS-1, -5, -9, -12, -15 and -18 gene promoters are hypermethylated in colorectal cancer, suggesting decreased expression of these enzymes in this state [23]. In addition, both ADAMTS-15 and -18 genes undergo frequent mutations in colorectal cancer cells, but no evidence has yet presented of their effect in expression or function of the enzymes. In contrast, ADAMTS-4 and -5 are upregulated in glioblastomas (GBMs), with a possible role in increased degradation of brevican thereby increasing invasive potential [24, 25]. Increased versican, which may be related to modulated ADAMTS expression, is also observed in canine colonic adenomas and carcinomas [26]. Versican but not decorin accumulation is related to malignancy in mammographically detected high density and malignant-appearing microcalcifications in non-palpable breast carcinomas [27].

To gain further insight into whether it is likely for the overexpressed versican to be degraded by ADAMTS as a cancer invasive mechanism, we examined ADAMTS-1, -4 and -5 expression in healthy and cancerous colon tissues and also in colon cancer cell lines of different metastatic potential. Since the aim of this study was to reveal potential targets for the development of novel not only anti-invasive but also anti-angiogenic therapies, the ADAMTS anti-angiogenic subgroup expression was also estimated. Additionally, since ADAMTS-1 anti-angiogenic effect is mediated by its thrombospondin motifs [11] and ADAMTS-20 contains 14 repeats of it, we also estimated its cellular levels in neoplastic and healthy colon.

## Materials and Methods

### Materials

Rabbit polyclonal antibodies, ab28284 (against N-terminal end of ADAMTS-1), ab84792 (against C-terminal end of ADAMTS-4), ab41037 (against 600–700 residues of ADAMTS-5) and ab60148 (against catalytic domain of ADAMTS-20) were purchased from Abcam (Cambridge, UK) and Goat anti-rabbit IgG conjucated with peroxidase was from EMD Millipore Corporation (Billerica, Massachusetts, USA). Mouse anti-tubulin (T9026) and peroxidase conjugated anti-mouse IgG (A4416) were obtained from Sigma-Aldrich Inc (St Luis, USA). Expose Mouse and Rabbit Specific HRP/DAB Detection IHC Kit (ab94710) was from Abcam. Total RNA was extracted from frozen tissue and cultured cells using the NucleoSpin Macherey-Nagel (Düren, Germany) extraction kit, following the manufacturer’s protocol. RT-PCR was obtained from Takara (Otsu, Shiga, Japan) One Step RT-PCR kit, used for fresh colon tissue and from Takara PrimeScript 1^st^ strand cDNA Synthesis kit and Finnzymes (Espoo, Finland) DyNAzyme II DNA Polymerase kit, used for cultured cells. Human Primers for all molecules were designed in the lab, using the PerlPrimer program. All other chemicals used throughout the study were of the best available analytical grade.

### Studies on cells

#### Cell cultures

Caco-2, DLD-1 and HT-29 colon cancer cell lines were purchased from the American Type Culture Collection (Manassas, VA). The aggressiveness of the cell lines is lower in Caco-2 cells and higher in HT-29 cells. DLD-1 and HT-29 cells were cultured in RPMI 1640 medium with 2 mM L-glutamine and supplemented with 10 mM HEPES, 1 mM sodium pyruvate, 4.5 g/L glucose, 1.5 g/L sodium bicarbonate and 10% fetal bovine serum (FBS), when required, as recommended by ATCC. Caco-2 cells were cultured in Eagle’s Minimum Essential medium with Earle’s BSS and 2 mM L-glutamine (EMEM) and supplemented with 1.0 mM sodium pyruvate, 0.1 mM nonessential amino acids, 1.5 g/L sodium bicarbonate and 20% fetal bovine serum when required, as also recommended by ATCC. Cells were cultured at 37°C, 5% CO_2_ and 100% humidity.

#### Cell lysis

The pellets from cultured cells were treated with appropriate lysis buffer, containing 25 mM Hepes, 150 mM NaCl and 5 mM EDTA in 10% glycerol and 1% Triton X-100. Extractions were performed for 30 min at 0°C, under vortexing for 4 sec every 10 min, with the presence of phosphotyrosyl phosphatase inhibitor Na_3_VO_4_ (50 mM) and of the following protease inhibitors: Aprotinin (1250 μg/mL), Pefablock (1000mg/mL), Leupeptin (10mM), following centrifugation in 10.000 rpm for 5 min.

### Studies on human samples

#### Patients

The cancerous colon tissues were obtained from patients (38 patients, mean age; 73, age range; 50–81), who underwent surgical operation due to colorectal carcinoma. Nine patients of stage A (Duke’s), thirteen patients of stage B, twelve patients of stage C and four patients of stage D were included in this study. The patients included in the study were free of other disease and never before suffered of any disease. Healthy colon tissues (N = 6) were also included in our study. This study design had been approved by the Ethical Committee of the University Hospital of Patras, Greece, and written informed consent was obtained from all patients entering the study.

#### Sequential extraction of extracellular and cell-associated components from tissues

The collected normal and cancerous colon tissues were diced and sequentially extracted with 10 vols of 10 mM disodium phosphate, 0.14 M NaCl, pH 7.4 (PBS), 4 M Guanidine Hydrochloride (GdnHCl)– 0.05 M sodium acetate pH 5.8 and 4 M GdnHCl-0.05 M sodium acetate-1% Triton X–100, in order to obtain the soluble and the membrane-bound forms of ADAMTSs. Extractions were performed for 24 h at 4°C under gentle shaking, with the presence of the following proteinase inhibitors: ε-amino-n-caproic acid (0.1 M), PMSF (0.4mM), N-ethylmaleimide (10mM), disodium EDTA (10mM) and benzamidine-HCl (5mM). The extracts were collected and stored at -20°C until use [28–30]. The extraction protocol was designed in such a way to separate ADAMTS existed in free forms in the tissues and should be extracted in PBS (associative conditions), from ADAMTS interacting with other extracellular macromolecules that should be extracted in 4M GdnHCl (dissociative conditions) and from ADAMTS existing in cell membranes that should be extracted especially in 4M GdnHCl-1% Triton X–100.

#### Immunohistochemistry

Immunohistochemistry (IHC) was performed on 5 μm sections cut from formalin fixed, paraffin-embedded blocks as described previously [29], using primary polyclonal antibodies against ADAMTS-1, -4, -5 and -20 diluted 1:6250, 1:2000, 1:650 and 1:6250 respectively, in TBS containing 1% (w/v) BSA. The obtained antigen-antibody complexes were visualized by incubation with Goat anti-rabbit HRP conjugate and the staining was developed with DAB/hydrogen peroxide of IHC Kit (Abcam) according to manufacturer’s instructions. Finally, the sections were counterstained with hematoxylin. Positive staining was scored according to the whole intensity of each one of the sections.

### Western blot analyses

About 20 μg of protein, quantified using Bradford assay (BioRad), from the tissues and cell cultures extracts, and the cell cultures media were precipitated with 5 vols of ethanol, dissolved in 20 μl of Laemmli sample buffer and applied to Polyacrylamide gel electrophoresis (PAGE) on gels of 10% concentration in acrylamide as previously described [28]. The macromolecules were then transferred to polyvinylidene difluoride (PVDF) membranes (Immobilon-P, Millipore) at a constant current of 80 mA at 4°C for 20 h in 0.05 M Tris/HCl, pH 8.3 and the membranes were washed with 0.14 M NaCl in 0.01 M phosphate buffer (PBS), pH 7.2, containing 0.1% (v/v) Tween-20 (PBS-T). After blocking with 5% bovine serum albumin (BSA) in PBS-T, the membranes were immersed in antibody solution against either ADAMTS-1, -4, -5 or -20 diluted 1:5,000, 1:500, 1:250 and 1:5,000 respectively, in PBS containing 1% bovine serum albumin (BSA) in PBS-T and incubated for 1 h at room temperature. After repeated washings with PBS-T, the membranes were incubated with proper secondary antibody diluted 1:10,000 in PBS-1% BSA, incubated for 1 h at room temperature and washed with PBS-T. The immunoreacting bands were visualized by enhanced chemiluminescence method (ECL) (Amersham, UK), according to the manufacturer's instructions and by exposure to Agfa Curix X-ray film, in time varied from 1 to 30 min, depending on the experiment.

### RNA extraction and RT-PCR analyses

Fresh colon tissues were pulverized in liquid nitrogen and as cultured cells were subjected to total RNA extraction, using the Nucleospin extraction kit, as described by the manufacturer’s instructions and treated with RNase-free DNase to remove contaminating genomic DNA. For colon tissues, strand cDNA was synthesized from 80 ng of total RNA in 50μl reaction components for one-step RT-PCR kit, according to the manufacturer’s instructions. This reaction mixture contained in addition 1μM of the sense and antisense primers shown in Table 1. The amplification was performed in a GeneAmp 2400 thermal cycler (Perkin-Elmer Co.) and the reaction profile used for all primers sets was: 95°C for 10 min for the activation of DNA polymerase and inactivation of reverse transcriptase and then 25–35 cycles, depending on the analysis, at 94°C for 30 sec, 52–58°C depending on the primer set for 1 min and 72°C for 1 min to finalize extension. For cell cultures, two step RT-PCR was applied and the first strand cDNA was synthesized from 1 μg of total RNA in 20 μl reaction components for PrimeScript 1^st^ strand cDNA Synthesis kit, using random 6mers, according to manufacturer’s instructions. Then 100 ng of cDNA were amplified in 50 μl reaction components for DNA Polymerase kit. This reaction mixture contained in addition 0.5 μM of the sense and antisense primers shown in Table 1. The amplification was performed in a GeneAmp 2400 thermal cycler (Perkin-Elmer Co.) and the reaction profile used for all primers sets was: 95°C for 2 min, 94°C for 30 sec, 52–58°C depending on the primer set for 1 min and 72°C for 1 min to finalize extension and was repeated for 25–35 cycles, depending on the analysis. The reaction products were separated by electrophoresis in 2% (w/v) agarose gels containing Gelstar Stain to visualize the amplified cDNA fragments under UV. The gels were then scanned and the bands were analyzed densitometrically. Quantitative differences between cDNA samples were normalized by including GAPDH in all experiments.

**Table 1:** Nucleotide sequence of the primers and Hybridization Temperature for each primer set used in RT-PCR experiments.

Type of primer	Nucleotide sequence	Hybridization Temperature	Size of product (bp)
*ADAMTS-1* Sense Antisense	GGACAGGTGCAAGCTCATCTG TCTACAACCTTGGGCTGCAAA	58	72
*ADAMTS-4* Sense Antisense	GACACTGGTGGTGGCAGATG TCACTGTTAGCAGGTGCGCTTTA	61	75
*ADAMTS-5* Sense Antisense	GTTTACTCGGGAGGATTTATGTGG GGAACCAAAGGTCTCTTCACAG	50	204
*ADAMTS-20* Sense Antisense	AATCGTCCTGAGCCAAGAAAGG GGAAGCCACCTCACATTAGAGG	52	182

### Statistical analysis

Normality of distribution of values was tested with Kolmogorov-Smirnov test. Results were statistically analyzed using the unpaired t-test to detect differences between groups. *P*≤0.05 was regarded as statistically significant.

## Results

### ADAMTSs expression in colon cancer cell lines

#### RT-PCR analyses

**Fig 1:**
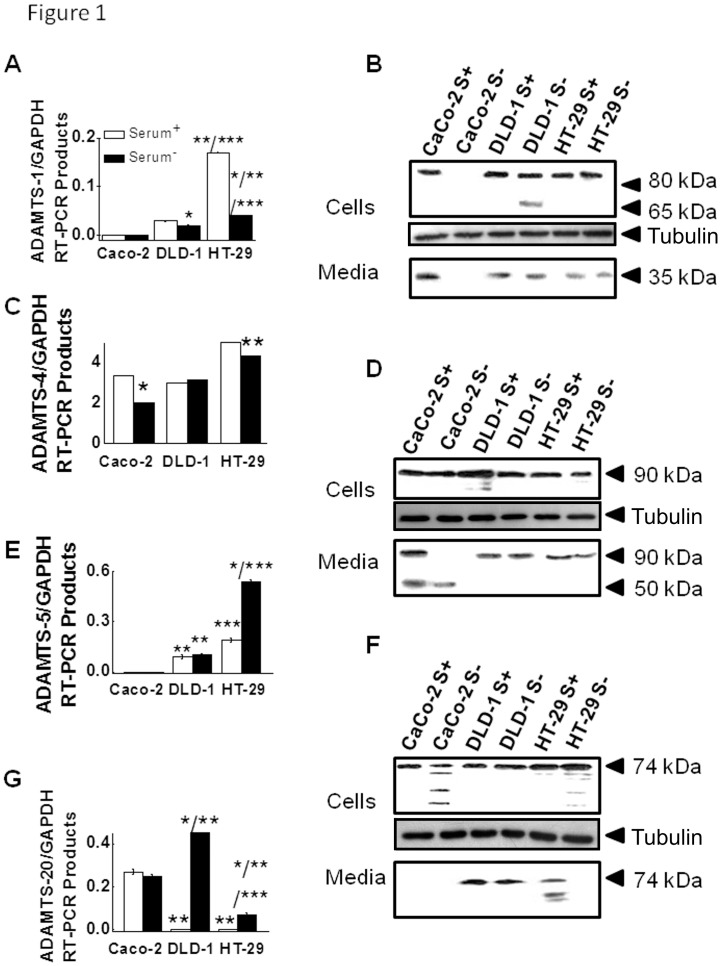
Expression of ADAMTSs in cancer cell lines. Semi-quantitative RT-PCR analysis of (A) ADAMTS-1, (C) ADAMTS-4, (E) ADAMTS-5 and (G) ADAMTS-20 in cultured cells (white/black column; presence/absence of serum). Values are the mean GAPDH-normalized ± S.D. (B) ADAMTS-1, (D) ADAMTS-4 and (F) ADAMTS-5 protein distribution in cell extracts and in media of cultured cells. (S^+/-^: presence/ absence of serum) *P≤0.05; statistically significant differences compared to cells cultured in the presence of serum. **P≤0.05; statistically significant differences compared to Caco-2 cells, ***P≤0.05; statistically significant differences compared to DLD-1 cells. For comparison, tubulin immunoidentification in cell extracts was included in B, D and F.

In order to investigate possible implication of ADAMTSs in CRC, we examined their expression at RNA and protein level in three colon cancer cell lines. The results indicated that in RNA level, ADAMTS-1 expression was slightly dependent on cancer cell aggressiveness; it was not found to be expressed in Caco-2, while it showed low expression in DLD-1, which was slightly increased in HT-29 cells. Moreover, in HT-29 cells cultured in the presence of serum, ADAMTS-1 expression was found elevated (Fig. 1A). On the contrary, high levels of ADAMTS-4 RNA were found in all three cell lines. However, ADAMTS-4 expression was found to be increased in an aggressiveness-related manner, especially when cells cultured in the absence of serum. Moreover, with the exception of DLD-1 cells, increased expression of ADAMTS-4 was observed when cells were cultured in the presence of serum (Fig. 1C). As shown in Fig. 1E, no ADAMTS-5 expression was found in Caco-2 cells, while it was mainly observed in HT-29 cells. Unlike, ADAMTS-1 and -4, ADAMTS-5 expression was found to be down-regulated in cells cultured in the presence of serum. A different expression pattern was observed for ADAMTS-20. As shown in Fig. 1G, in DLD-1 and HT-29 cells, it was found to be expressed only when cultured in the absence of serum. However, serum presence did not seem to influence ADAMTS-20 expression in Caco-2 cells.

#### Western Blot analyses

ADAMTS-1 active form was detected in DLD-1 and HT-29 cell lines, as a band of 80 kDa in the cell extracts, while only fragments of ADAMTS-1 of 35 kDa were detected in the media (Fig. 1B), regardless of the presence of serum. These fragments could be products of either autocatalytically processed ADAMTS-1 or catalytically processed ADAMTS-1 by MMPs. Given the fact that such fragments were detected only extra- and not intra-cellularly, they are more possible resulting from MMPs activity. In Caco-2 cell lines this metalloprotease was only detected in the presence of serum at same molecular form as in both other cell lines (Fig. 1B). An additional band of 65 kDa was observed in the extracts of DLD-1 cells cultured in the absence of serum.

ADAMTS-4 active form was detected in DLD-1 and HT-29 cell lines and in the presence or not of serum, as a band of 90 kDa, intra- and extracellularly (Fig. 1D). In the case of Caco-2 cells, the 90 kDa band was only detected in cell extracts, whereas in cell media an extra immunoreactive band of 50 kDa was observed, probably as a result of its own autocatalytic activity (Fig. 1D).

ADAMTS-5 active form was detected as a band of 74 kDa, in all three cell lines cultured under any conditions (Fig. 1F). Additional bands of various sizes, smaller than that of the active form, were observed in the cell extracts of HT-29 and mainly of Caco-2 cells cultured in the absence of serum. Interestingly, no immunoreactive band of any size was detected in the media of any of these cell cultures. However, ADAMTS-5 active form was also detected extracellularly, in HT-29 cells cultured in the presence of serum and in DLD-1 cell lines. Moreover, in HT-29 cells cultured in the presence of serum, additional bands of smaller sizes were also detected extracellularly, suggesting a posttranslational truncation of the enzyme resulting from either its own autocatalytic activity or MMPs activity.

In contrast to all other ADAMTSs studied, ADAMTS-20 protein was not detected in these three colon cancer cell lines.

### In situ expression of ADAMTS-1, -4, -5 and -20 by IHC

**Fig 2:**
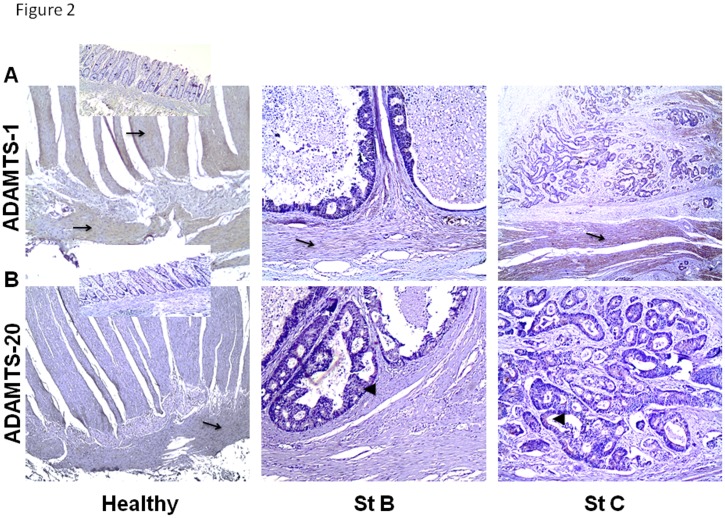
Immunohistochemical localization of ADAMTS-1 and ADAMTS-20 in healthy and in cancerous colon. Healthy colon tissue and cancerous colon of stage B and C, stained for ADAMTS-1 (A) and ADAMTS-20 (B). (A, magnification; x4, anatomic site; cecum, magnification; x4, anatomic site; cecum, magnification; x4, anatomic site; rectum and B, magnification; x4, anatomic site; cecum, magnification; x20, anatomic site; cecum, magnification; x20, anatomic site; rectum). Insets of A and B (magnification of all; x10) show the Mucosa and the Submucosa layer. Arrows in healthy colon show the expression of ADAMTS in the muscle tissue. Arrows in cancerous colon show the expression of ADAMTS in stroma, while arrowheads show the expression of ADAMTS in cancer cells.

**Fig 3:**
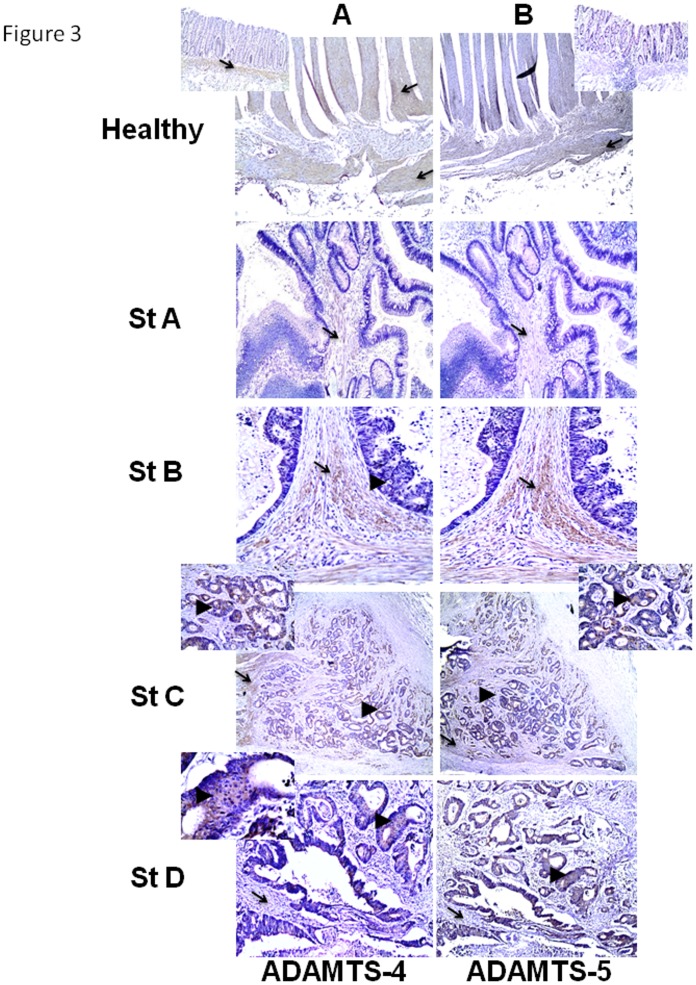
Immunohistochemical localization of ADAMTS-4 and ADAMTS-5 in healthy and in cancerous colon. Healthy colon tissue and cancerous colon of stage A, B, C and D, stained for ADAMTS-4 (A) and ADAMTS-5 (B). (A, magnification; x4, anatomic site; cecum, magnification; x10, anatomic site; cecum, magnification; x20, anatomic site; cecum, magnification; x4, anatomic site; rectum, magnification; x20, anatomic site; rectum and B, magnification; x4, anatomic site; cecum, magnification; x10, anatomic site; cecum, magnification; x20, anatomic site; cecum, magnification; x4, anatomic site; rectum, magnification; x10, anatomic site; rectum), stained for ADAMTS-5. Insets of A show the Mucosa and Submucosa layer on the healthy colon (magnification; x10) and the cytoplasmic localization of ADAMTS-4 in cancer cells of stage C and D (magnification; x20 and x40, respectively). Insets of B show the Mucosa and Submucosa layer on the healthy colon (magnification; x10) and the cytoplasmic localization of ADAMTS-5 in cancer cells of stage C (magnification; x20). Arrows in healthy colon show the expression of ADAMTS in the muscle tissue. Arrows in cancerous colon show the expression of ADAMTS in stroma, while arrowheads show the expression of ADAMTS in cancer cells.

ADAMTS-1, -4, -5 and -20 in situ expression and localization was next analyzed in colon tissue sections from paraffin-embedded blocks, using the appropriate antibodies. Strong staining for ADAMTS-1 was observed in healthy colon, mainly in the muscular layers and in the connective tissue between the two layers (Fig. 2A, Healthy). In CRC, ADAMTS-1 showed strong expression in muscle tissue (arrow in Fig. 2A, St. C) and lower mainly cytoplasmic expression in cancer cells of stage C specimens. Reduced or no staining for ADAMTS-1 was observed in muscle tissue of stage B specimens (Fig. 2A, St. B) and in specimens of other stages (C and D), respectively. Staining for ADAMTS-4 in healthy colon tissue was observed in the two layers of Muscularis Externa and in the Submucosae (Fig. 3A, Healthy). In early cancer stages, stages A and B, ADAMTS-4 exhibited a similar expression pattern to ADAMTS-1 since it showed staining in stroma (arrows in Fig. 3A, St. A, St B) and quite fainter or no staining in cancer cells (arrowheads in Fig. 3A St. B and St. A, respectively). However, in specimens of stages C and D, cancer cells showed considerable cytoplasmic expression of ADAMTS-4 (arrowheads in Fig. 3A St. C and St. D and their insets, respectively). Moreover, less or no expression of ADAMTS-4 was observed in stroma cells in specimens of stages C and D, respectively (arrows in Fig. 3A St.C and St. D, respectively). As for ADAMTS-5, quite low staining was observed in the longitudinal layer of Muscularis Externa in healthy colon specimens (arrow in Fig. 3B, Healthy). In CRC, no staining for ADAMTS-5 was observed either in stroma or in neoplastic epithelial cells, of stage A specimens (arrow and arrowhead in Fig. 3B, St.A, respectively), while expression of ADAMTS-5 was detected in stroma cells of stage B specimens (arrow in Fig. 3B, St. B). As ADAMTS-4, ADAMTS-5 also exhibited strong cytoplasmic staining in cancer cells of stage C and D specimens (arrowheads in Fig. 3B St.C and St. D, respectively) and no stromal expression in stage D. Finally, ADAMTS-20 also exhibited low staining in the longitudinal layer of Muscularis Externa in healthy colon specimens (Fig. 2B, Healthy), while in CRC it was detected only in cancer cells of stage B and mainly of stage C specimens (arrowheads in Fig. 2B, St.B and St. C, respectively).

### ADAMTSs expression in colon cancer tissues

#### RT-PCR analyses

**Fig 4:**
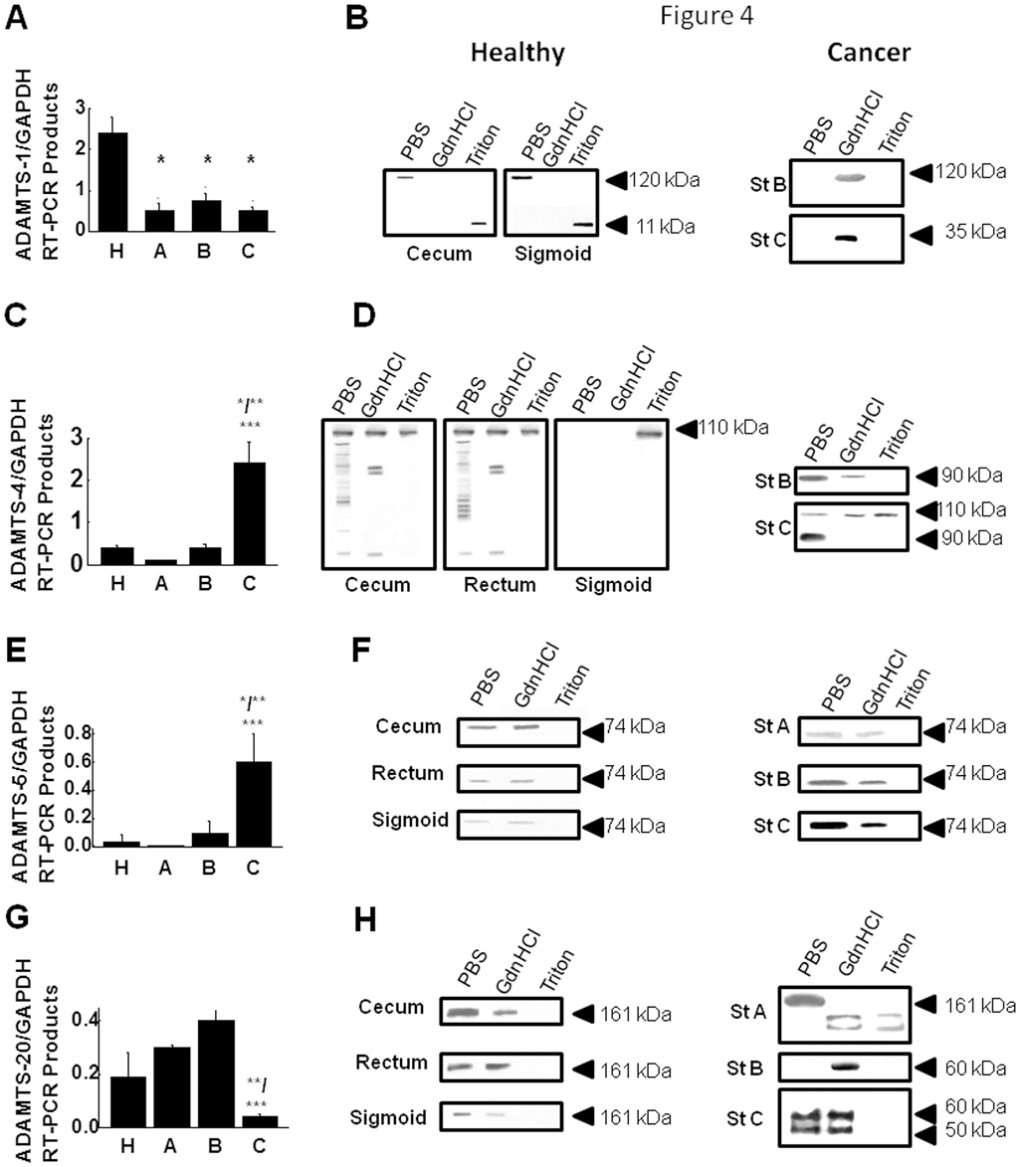
Expression of ADAMTSs in healthy and cancerous colon tissues. Semi-quantitative RT-PCR analysis of (A) ADAMTS-1, (C) ADAMTS-4, (E) ADAMTS-5 and (G) ADAMTS-20. Values are the mean GAPDH-normalized ± S.D. Protein distribution of (B) ADAMTS-1, (D) ADAMTS-4, (F) ADAMTS-5 and (H) ADAMTS-20. *P≤0.05; statistically significant differences compared to healthy tissues. **P≤0.05; statistically significant differences compared to stage A. ***P≤0.05; statistically significant differences compared to stage B. Healthy Cecum, Rectum and Sigmoid colon tissues are shown; *A-C*: Duke’s CRC stages. Arrows indicate the migration of molecular mass markers. PBS, GdnHCl and Triton are the sequential extracting solutions used (see text).

RNA levels of ADAMTSs were examined in healthy cecum, sigmoid and rectum. Of all proteases investigated, ADAMTS-1 showed the highest expression (more than double to the internal control-GAPDH), while ADAMTS-5 was the less expressed protease (almost at zero level) (data not shown). RT-PCR analyses indicated that *ADAMTS-1* was down-regulated in cancer specimens of any stage compared to the healthy colon, with the greatest decrease to about 20% in stage A specimens (Fig. 4A). These data are in agreement with previous studies, where *ADAMTS-1* had been found to be down-regulated in many types of cancer [21, 22]. A different expression pattern from *ADAMTS-1* was observed for *ADAMTS-4* and-*5*, which were found to be overexpressed in stage C specimens; almost 14-fold and 20-fold compared to healthy colon tissue, respectively (Fig. 4C, E). These data were in agreement with previous studies in other types of cancer, where ADAMTS-4 and -5 had been found to be over-expressed in order to degrade ECM proteoglycans, such as agreecan and versican to facilitate cancer cell invasion [24, 25]. Following the same experimental procedure for *ADAMTS-20*, it was found elevated in stage A and B specimens compared to the healthy tissues by 50% and 100%, respectively, but in stage C specimens it exhibited a decrease to 20% (Fig. 4G).

#### Western Blot analyses

ADAMTS-4, -5 and -20 were detected in cecum, sigmoid and rectum, whereas ADAMTS-1 was detected only in cecum and sigmoid. However, they differed in their extractability revealing different types of interactions and/or localization of these enzymes within the colon tissue.

ADAMTS-1 was detected only in PBS and GdnHCl/Triton extracts of healthy cecum and sigmoid, as a band of 120 kDa and 11 kDa respectively, representing the latent form of the enzyme and products of uncompleted synthesis, while none of the extracts contained the active form (80 kDa) of the enzyme. (Fig. 4B). ADAMTS-1 latent form was also detected in cancerous cecum of stage B, using dissociative conditions showing that it interacted with ECM components, such as TIMP-3 or a2- macroglobulin, which maintained the enzyme in its inactivated form. In cancerous rectum of stage C it was detected in GdnHCl extracts as a fragment of 35 kDa, possibly resulting from MMPs activity (Fig. 4B). A different figure was obtained from sigmoid, where ADAMTS-1 was not detected in any cancer stage. Thus, it could be claimed that ADAMTS-1 role in CRC depends at a large extent on the anatomic site.

To continue, the latent form of ADAMTS-4 was detected in Triton extracts of all three anatomic sites of healthy colon as a band of 110 kDa. However, in PBS and GdnHCl extracts of both cecum and rectum, the active form of the enzyme (90 kDa) and multiple fragments of various molecular sizes and two distinct fragments of 60 kDa and 70 kDa were detected, respectively (Fig. 4D). On the contrary, ADAMTS-4 was detected in stage B and C specimens mainly in its active form. In stage B cecum specimens, the active form of the enzyme was detected in PBS and GdnHCl extracts, while in stage C rectum specimens, except for the active form which was found in PBS extracts, an additional band of 110 kDa, representing its latent form, was also detected in all tree extracts.

Following the same experimental procedure, ADAMTS-5 active form was observed in PBS and GdnHCl extracts of healthy colon but also of all cancer stages, however, with a stage-related increased immunoreactivity (Fig. 4F).

Finally, western blot analysis revealed the presence of latent form of ADAMTS-20 (161 kDa) in the PBS and GdnHCl extracts of the three anatomic sites of healthy tissues, sigmoid being containing the lowest amounts. Interestingly, it was also detected in all cancer stages, however with differences in its extractability and in the molecular forms. In stage A sigmoid specimens, the latent form of the enzyme was detected in PBS extracts, indicating that it existed rather freely in that tissue. ADAMTS-20 fragments were detected in GdnHCl and GdnHCl/Triton extracts (Fig. 4H). In GdnHCl extracts of stage B cecum specimens, a single fragment of 60 kDa was obtained, while in PBS and Gdn-HCl extracts of stage C rectum specimens, an additional smaller fragment of 50 kDa was obtained.

## Discussion

Accumulating evidence of ADAMTSs implication in human malignancies has demonstrated their significant role in tumor progression [21, 24, 25]. Over-expression of these proteases is consistent with the requirements of carcinoma cells to remove proteoglycans of ECM, such as versican and aggrecan, as a complementary mechanism to collagen degradation by collagenases and gelatinases. Accordingly, increased expression of versican has been observed in neoplasias of colon and rectum [26, 30]. Additional significant information would give a study in ADAMTSs expression in CRC, since a potential degradation of versican would result in active versican fragments with established roles in tumor progression [14]. On the other hand, some members of ADAMTSs family, those reported to have potential anti-angiogenic role, have been found epigenetically silenced in various types of cancer, including the CRC [12]. In this study, a possible modulation of ADAMTS-1, -4, -5 and -20 expression and distribution in colon cancer compared to healthy colon was investigated. The experimental findings revealed a similar expression pattern for ADAMTS-4 and -5 and a completely different expression pattern for ADAMTS-1 and -20.

### ADAMTS-4 and -5 are over-expressed in CRC

The results of this study support the notion that ADAMTS-4 and -5 are over-expressed in CRC possibly for tissue disruption that would facilitate cancer cell invasion and metastasis. However, slight differences between ADAMTS-4 and -5 expression in CRC were observed. RT-PCR and western blotting analyses in cultured cells of three different cancer cell lines revealed that expression levels of ADAMTS-5 were stronger related to cancer aggressiveness, as compared to ADAMTS-4. More specifically, extracellular ADAMTS-5 active form was not detected in Caco-2 cells, in contrast to ADAMTS-4 whose extracellular active form was detected in all three cell lines, regardless of cell aggressiveness. In addition to this, extracellular fragments of ADAMTS-4 and -5, possibly resulting from their own autocatalytic activity [31], were detected in both the Caco-2 and HT-29 cell lines. Hence, it could be suggested that among these two enzymes, ADAMTS-5 was over-expressed mainly in mediate/highly-aggressive cancer cells, while ADAMTS-4 performed a wider range of expression, regardless of cancer aggressiveness. Moreover, *ADAMTS-4* and-*5* transcription activity was found to be differentially regulated by the presence of serum. This finding most probably suggests that ADAMTS-4 up-regulation was possibly mediated via inflammatory cytokines and growth factors, and data from studies in osteoarthritis support this [32]. On the contrary, ADAMTS-5 down-regulation was possibly mediated by growth factors, such as FGF-2 [33]. However, serum seemed to be crucial for secretion of the active form of ADAMTS-4 and -5 in both the Caco-2 and the HT-29 cells.

ADAMTS-4 and -5 also exhibited the same localization pattern. In healthy colon, both enzymes, but mainly ADAMTS-4, were expressed in muscle tissue. In CRC, the expression levels of ADAMTS-4 and -5 were relatively decreased in early cancer stages (A, B) and the localization of the metalloproteinases was primarly at stroma cells. Interestingly enough, in late cancer stages (C, D) their expression levels were augmented and the examined enzymes were located mainly in malignant cells.

Finally, ADAMTS-4 and -5 displayed similar expression pattern during cancer progression. They were both over-expressed at stage C, that is characterized by lymph node metastasis. Hence, it is possible that ADAMTS-4 and mainly ADAMTS-5 play a key role in tumor progression to higher stages of CRC by degrading ECM, so as to facilitate cancer cell invasion, in a similar manner as it has been previously demonstrated for hyaluronidase [34]. Western blot analysis also confirmed the presence of active forms of both ADAMTS-4 and -5. In contrast to healthy colon, where ADAMTS-4 was fragmented, in CRC it was present mainly in its active form. This observation was in accordance with previous studies suggesting that fragments of ADAMTS-4 had an anti-metastatic role, in contrast to the active form of the enzyme which usually exhibits a pro-metastatic function [35]. However, immunobloting also revealed differences between ADAMTS-4 and -5. Apart from its active form, ADAMTS-4 was also present in its latent form, while ADAMTS-5 was constantly present in its active form during cancer progression. Taking these data into account, it could be suggested that ADAMTS-4 only partially contributes to CRC progression. On the other hand, ADAMTS-5 serves as the cardinal component activation of which, fires CRC progression to higher stages. Notably, this finding is in harmony with previous studies having demonstrated a similar role of ADAMTS-5 in laryngeal cancer [29]. Hence, future studies should focus on further investigating the mechanisms of ADAMTS-4 and -5 activation and to what extent these two enzymes are specialized in cleaving the modified substrates/proteoglycans which are present in CRC.

### ADAMTS-1 and ADAMTS-20 are down-regulated in CRC

The different expression levels of *ADAMTS-1* and-*20* in CRC, despite their individual differences, further differentiate their implication in cancer, as compared to ADAMTS-4 and -5. ADAMTS-20 down-regulation, with the exception of Caco-2 cells where it is still expressed, was achieved in transcription level by serum components but mainly in post-transcription level, since no protein is being produced. On the contrary, despite the low transcriptional activity of *ADAMTS-1*, the catalytically active enzyme was being produced but it was not secreted by the cells. ADAMTS-1 fragments found in the media of cell cultures were possibly products of catalytically processed ADAMTS-1 by MMPs. As a result, the removal of ADAMTS-1 from cell membrane limits its anti-angiogenic properties [36].

In healthy colon ADAMTS-1 and -20 performed the same localization, since they were both expressed in muscle tissue, though ADAMTS-1 showed much higher expression than ADAMTS-20. Interestingly, in CRC they were expressed by different cell types. ADAMTS-1 was mostly expressed by stroma cells, in contrast to ADAMTS-20 which was expressed by cancer cells. ADAMTS-1 expression by stroma cells could be an attempt to arrest the progress of cancer by inhibition of angiogenesis; an attempt finally failed as western blot analysis revealed by the presence of enzyme fragments in stage C. ADAMTS-20 expression by cancer cells could be a result of cross-talking between cancer cells and stroma cells. Given the fact that no ADAMTS-20 is being produced by cultured colon cancer cells, in contrast to colon cancer tissue, where the enzyme is being produced, as it was confirmed by both IHC and Western blot, it could be said that tumor micro-enviroment plays an important role in ADAMTS-20 expression. However, as Western blot analysis revealed the latent form of the enzyme, which was present in healthy colon, is being produced only in early cancer stage while in late cancer stages the enzyme is being fragmented. These fragments are freely circulated but also in association with ECM components; the exact role of these fragments though remains unknown. Taking together these data, it could be said that although ADAMTS-1 and -20 are both generally down-regulated in CRC, their low expression came from stroma or stroma-induced cancer cells, respectively, where they were present as fragments.

## Conclusions

During cancer progression, a reorganization of the extracellular matrix takes place to influence cellular proliferation and invasion. ADAMTSs are key molecules in this event, since they are extracellular proteases, possessing also anti-angiogenic activity. From the results of our study in CRC tissue and cells, we conclude that ADAMTS-4 and -5 expression positively correlates with cancer progression, whereas the anti-angiogenic ADAMTS-1 and -20 were found to be down-regulated and degraded. Thus, our results provide a mechanism of CRC progression and invasion mediated by specific ADAMTS members.
